# Effects of a Web-Based Parent–Child Physical Activity Program on Mental Health in Parents of Children with ASD

**DOI:** 10.3390/ijerph182412913

**Published:** 2021-12-07

**Authors:** Mengxian Zhao, Yonghao You, Shihui Chen, Linlin Li, Xiru Du, Yongtai Wang

**Affiliations:** 1 School of Physical Education, Shenzhen University, Shenzhen 518060, China; zhaomengxian@szu.edu.cn; 2 Department of Physical Education, Institute of KEEP Collaborative Innovation, Shenzhen University, Shenzhen 518060, China; 3 Department of Sports Science, Hefei Normal University, Hefei 230061, China; hao2703@hfnu.edu.cn; 4 Department of Kinesiology, Texas A&M University, Texarkana, TX 75503, USA; 5 School of Sports Social Science, Shandong Sport University, Jinan 250102, China; lilinlin@sdpei.edu.cn; 6 College of Sport Arts, Guangzhou Sport University, Guangzhou 510500, China; 7 College of Health Sciences and Technology, Rochester Institute of Technology, Rochester, NY 14623, USA; ytwchst@rit.edu

**Keywords:** mental health, depression, anxiety, autism, parent–child dyadic approach, web-based physical activity, social interaction, quality of life

## Abstract

Parents of children with ASD experience a higher incidence of mental health difficulties, including stress, depression, and anxiety, than parents of children without ASD. According to studies related to ASD, parent–child physical activity programs are an effective approach to encourage both parents and their children with ASD to exercise together, thus improving the mental health of parents due to this interactive family activity. The purpose of the present study was to explore the effects of this web-based parent–child physical activity program on the mental health of parents of children with ASD. A total of 94 parent–child pairs consented to participate in this study, and 75 parent–child pairs completed the study. Three instruments—DASS-21, PSI-4-SF, and WHOQOL-26—were used to measure mental health, parental stress, and quality of life, respectively. A randomized controlled trial design was implemented to examine the effectiveness of the 10-week web-based parent–child physical activity program on improving the mental health of parents of children with ASD. The results showed that after the 10-week parent–child physical activity program, there were significant differences in overall DASS-21 and PSI-4-SF for the experimental group, compared with control group (*p* < 0.05), which indicated that the parent–child physical activity program has a positive influence on mental health in parents of children with ASD. One sub-area of WHOQOL-26 between the experimental and control groups across pre-/post-testing intervals also showed greater reductions in the item of psychological health (*p* < 0.05). In conclusion, the findings demonstrated the efficacy of the web-based parent–child physical activity program for improving mental health in parents of children with ASD.

## 1. Introduction

Autism spectrum disorder (ASD) is a neurodevelopmental disorder characterized by impairments in social communication and restricted or repetitive behaviors [1]. The prevalence of ASD has experienced rapid growth over the past decade [2], and the situation has also been serious in other countries, making it a global public health problem. The pervasive impairments always appear in early childhood and endure across the child’s lifespan, seriously influencing physical and mental health and the quality of life of families with children with ASD [3].

The parents of children with ASD also experience mental health challenges, which refer to their thoughts, emotional regulation, and behaviors reflecting the psychological or biological processes underpinning their mental functioning [4]. It has been found that parents of children with ASD experience higher incidence of mental health difficulties—such as stress, depression, anxiety, and parental distress—than parents of children who are typically developing or with other types of disabilities [5,6,7,8,9].

Studies have reported that mental health deficits in such parents are considerably high, due to fears, insecurities, and worries about their children’s life, and there exists an association between the stress and anxiety levels of parents and overall ASD severity [10,11]. In addition to the core impairments of ASD, co-morbid difficulties, such as depression, disruptive behavior, hyperactivity disorder, and sleep disorder, have also been found to be associated with higher stress and anxiety in parents [12,13]. Therefore, the challenges relating to caring for children with ASD can lead to difficulties for parents, in terms of meeting their own healthy psychological state and quality of life [14,15,16]. Furthermore, their poor mental health may have negative effects on supporting and caring for their children with ASD [17]. Additionally, the parents of children with ASD face more difficulties in China, such as delayed diagnosis, limited early interventions (as the associated research started relatively late), and the majority of parents have to be responsible for the treatments by themselves, with insufficient training, guidance, and support [18,19], which may add to their psychological burden. These parents are always at high risk for caregiver syndrome, which poses a great risk to the health-related quality of life of parents, as well as the whole family [20].

The mental health problems of parents of children with ASD might be severely magnified during the COVID-19 pandemic. Therefore, these parents may require more mental health support and assistance [21]. With increased attention on children with ASD, many physical activities, as alternative treatments, have been shown to be effective for the remediation of behaviors of children with ASD. Some researchers have begun to focus on the psychological health of parents of children with ASD over the past decade, and evidence has shown that some interventions have a certain effect on improving mental health, such as cognitive behavioral therapy [22,23], expressive writing [24], mindfulness training [25], and relaxation therapy [26]. Although there are currently few studies that have been conducted directly on parents of children with ASD, the studies related to this area are still limited, especially studies using randomized controlled trials as intervention.

Physical activity, as an essential component of a healthy lifestyle, has become an increasingly important issue in health promotion, and many studies have demonstrated that physical activity interventions can be used as an effective treatment for improving mental health [27,28,29]; evidence has shown that children with ASD are still less physically active than those who are developing typically, due to their disabilities. It is also well known that the levels of physical activity of parents of children with ASD are low, indicating that there is a clear need to promote physical activity within the family unit [30].

As mentioned above, improving the mental health and wellbeing of the parents enhances the potential of the child with ASD to achieve a better quality of life [31,32], and parental involvement in treatments has been reported for its strengths and effectiveness [33]. The parent and child co-participation in physical activity is important for family interpersonal dynamics, including increased parent–child communication, enjoyment of physical activity, and improvement in their mental health [34]. In addition, during the COVID-19 pandemic, web-based activities, meetings, and gaming through the internet have become a popular support approach and is more beneficial and convenient for children with ASD and their parents in a family-based environment.

Parent–child physical activities could be a useful approach to involve both parents and their children with ASD to exercise together, and the fun and joyful time during parent–child physical activity not only brings family together, but also motivates inactive children with ASD to get actively involved and allows both the children and parents to cope with stress [35,36]. To date, there has been a lack of studies on the mental health issues of parents of children with ASD using parent–child dyadic web-based interventions. Therefore, the current study explores the effects of a web-based parent–child physical activity program on the mental health of parents of children with ASD.

## 2. Materials and Methods

### 2.1. Participants

**Figure 1:**
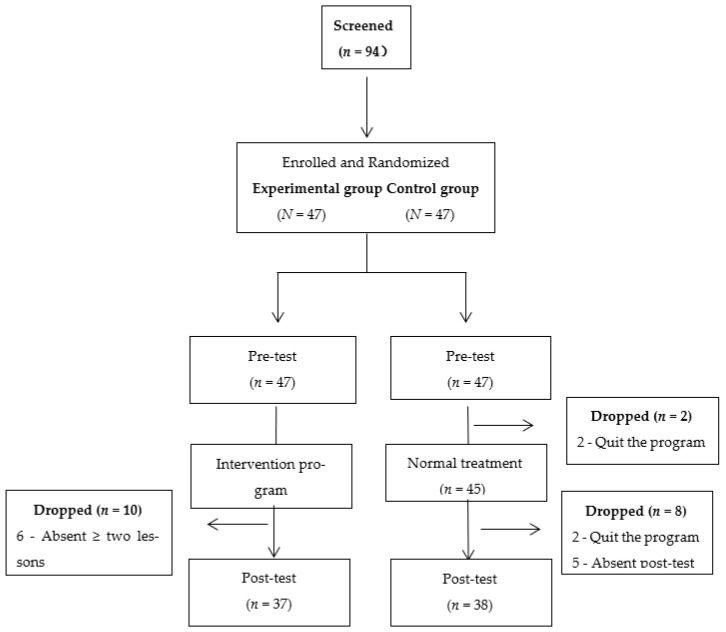
Participant selection process flow diagram.

Children with an average age of 6 years old were recruited through special schools, autism treatment centers, and community clinics in Beijing and Jinan, China in 2020. One of their parents (average age of 33.6 years old) was paired to participate in this intervention. Written informed consent was obtained from their parents before participating in this study. Ethics approval was granted by the Institutional Review Board of the University of the first author. The inclusion criteria for selecting participants were as follows: (a) availability of one parent and one child, (b) children’s age was between three and ten years old, and (c) children were diagnosed with ASD based on DSM-V criteria. A total of 94 parent–child pairs (comprising one parent and one child per pair) consented to participate in and completed the baseline survey. Forty-seven pairs of participants were randomly assigned to the experimental group, in which a web-based parent–child physical activity program was implemented as the intervention group. The other forty-seven parent–child pairs participated in regular activities as the control group. Figure 1 presents a flowchart of the participant selection process.

During the study, eight parent–child pairs withdrew from the program, six parent–child pairs were absent for more than two sessions, and five pairs were absent from the post-test. Therefore, a total of 75 participants (*n* = 75) completed the study. An independent sample *t*-test was conducted to compare the baseline data between the experimental group and control group. There were no significant differences between the two groups in terms of the demographic variables of participants, as listed in Table 1 (*p* > 0.05).

Table 1 also details the demographic characteristics of parents of children with ASD. There were 22 fathers and 53 mothers involved in the program. Most of families participating in the study had only one child. Among the parents, 50 out of 75 participants were housewives/househusbands.

### 2.2. Instruments

Mental health: The Depression Anxiety and Stress Scale (DASS-21), as a measure of mental health, is commonly used in current research. The scale is a 21-item, self-reported, norm-referenced measurement designed to investigate the severity of common depression, stress, and anxiety symptoms, [37] and Chinese versions have been shown to have excellent psychometric properties across normative and clinical samples and, importantly, with parents of youth with ASD [38,39]. Moreover, the response to treatment can be also measured. The scale has three sub-scales (depression, stress, and anxiety), with seven items in each sub-scale. In our sample, the Cronbach’s alpha for pre- and post-tests were 0.944 and 0.927.

Parenting stress: The Parenting Stress Index-Fourth Edition-Short Form (PSI-4-SF) is an abbreviated and improved version of PSI [40], which is one of the most commonly used measures of parenting stress in both clinical and research fields [41]. The PSI-4-SF is a 36-item, self-reported measure with three domains: Parental distress, parent–child dysfunctional interaction, and child difficulty. It has been used to measure the parenting stress of parents in high-risk populations [42,43], and to measure treatment effectiveness [44]. PSI-SF has been translated into different languages. The Cronbach’s alpha for pre- and post-tests were 0.885 and 0.892.

Quality of Life: The World Health Organization Quality of Life Assessment-26 (WHOQOL-26) is a short version of the WHOQOL-100 scales [45]. It is a widely used, reliable international instrument that consists of five sub-scales to measure the satisfaction of participants with different aspects of their life. It comprises four dimensions of QOL (physical, psychological, environmental, and social domains), and each item adopts a five-point assessment. The Cronbach’s alpha for pre- and post-tests were 0.861 and 0.877.

### 2.3. Intervention Procedures

A randomized controlled trial design was implemented to examine the effectiveness of the 10-week web-based parent–child physical activity program on mental health in parents of children with ASD. We adopted a pre-/post-test approach to monitor the progressive changes at two intervals on mental health throughout the 10-week intervention. Baseline data were collected one week before the intervention began, and the post-test was conducted one week after the 10-week program.

The intervention was a web-based parent–child physical activity program developed through the online curriculum from Youth Sport, which includes various physical educational programs based on the international physical activity guidelines and recommendations. The program involved the parents and children with ASD to participating together, and the current intervention was a structured physical activity program lasting 10 weeks, twice a week, for a total of 20 sessions, and each session lasted approximately 60 min. Each session was comprised of four parts: (a) warm-up activities; (b) parent–child exercises; (c) play-based exercises; and (d) cool-down and reward activities. There were three five-minute intervals for each session. All the parent–child pairs completed the same routine exercises under the guidance of trainers who were physical education teachers that were responsible for providing instructions, resolving difficulties, and facilitated practice for the parent–child dyad.

During the 10-week intervention program, all families in the experimental group received parent–child exercise training through a web-based curriculum. The curriculum consisted of teaching videos, live exercises, video games, and workshops. Each parent–child pair had a login account for the online curriculum, and the information was sent to the parents before the intervention. Furthermore, there was a chat group in WeChat, in which the parents could communicate and discuss how to better interact with their children and promote parent–child exercises. During the program, the control group participated in their family’s regular activities as normal. After the 10-week web-based parent–child physical activity program ended, the control group was given the same opportunity to participate in the parent–child physical activity program for ethical consideration.

The content of the parent–child physical activity program was designed especially for children with ASD and their parents, based on the impairment and characteristics of children with ASD [46]. Moreover, the program incorporated teaching videos with instructions and demonstrations, guiding the parents to engage in physical activities with their children. Meanwhile, each session contained a number of structured game elements, such as points, a level system, ranking, and a scoreboard for tracking progress [36]. To encourage the participants and make the role modeling effective, live courses were also implemented in the intervention. The participants could see other parent–child pairs through the online courses and interact with them through Zoom or Tencent Live. Table 2 describes the contents of the online parent–child physical activity program.

### 2.4. Data Collection and Analysis

In the current study, data was collected using a quantitative method to examine the effects of the online parent–child physical activity program on the mental health of parents of children with ASD, conducted both before (pre-test) and after (post-test) the intervention. The repeated measure MANOVA was used to analyze the main effect and interaction effect. The changes in overall and subdomains of DASS-21, PSI-4-SF, and WHOQOL-26 data were also analyzed to determine the differences between pre- and post-tests regarding mental health, parenting stress, and quality of life. The statistical analyses were conducted according to the P value, effect size, and sample power, and significance was two-tailed at *p* < 0.05.

## 3. Results

### 3.1. Effects of the Web-Based Parent–Child Physical Activity Program on Mental Health

#### 3.1.1. Effects of the Online Parent–Child Physical Activity Program on DASS-21

The Depression Anxiety and Stress Scale (DASS-21) was employed to evaluate the mental health of parents of children with ASD. As shown in Table 3, there were significant differences in terms of time: F = 147.937, *p* < 0.01, and time x group interaction: F = 106.032, *p* < 0.01. The results of the multivariate tests showed that the time factor had significant effects (*p* < 0.01) on DASS-21 over time, and the interaction between testing intervals (pre-test and post-test) and groups (experimental and control groups) also showed a statistically significant difference (*p* < 0.01). The mean scores of overall DASS-21 were similar between the two groups at baseline (*p* > 0.05) and indicated that there was no significant difference between the experimental group and the control group before the intervention. After the 10-week parent–child physical activity program, there were significant differences in the item of mental health between the experimental group and control group (*p* < 0.05). When compared with the control group itself, a significant improvement (*p* < 0.01) was found in overall DASS-21 for the experimental group. These results indicated that the parent–child physical activity program had a positive influence on the mental health of parents of children with ASD.

We also evaluated whether the online physical activity program demonstrated an effect in the three sub-areas of DASS-21 between the experimental and control groups over time. Before the intervention, no statistically significant differences were found between the experimental group and control group, in terms of stress, anxiety, and depression. After the 10-week web-based physical activity program, greater changes were observed in the items of stress, anxiety, and depression for the experimental group over time, compared with control group (*p* < 0.05), as shown in Table 4. These results showed that the program significantly reduced the scores in the sub-areas of DASS-21.

**Figure 2:**
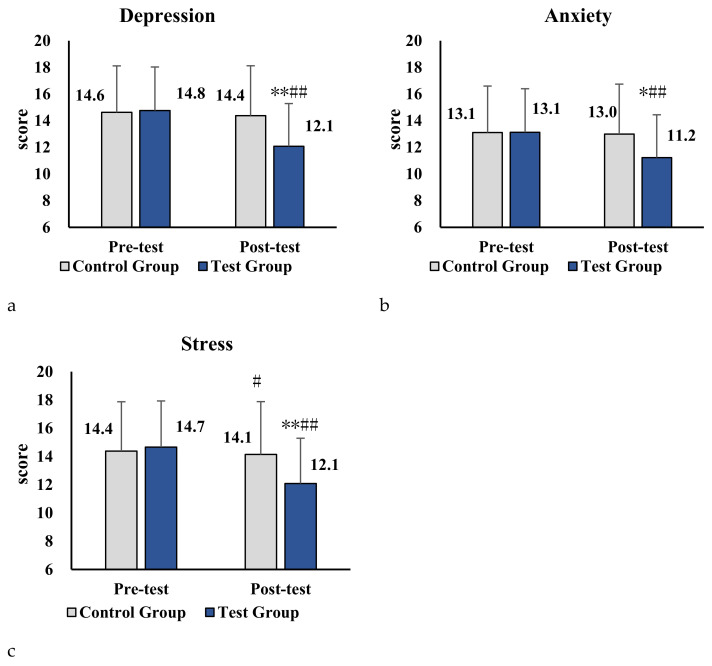
(**a**–**c**) Impact of parent–child physical activity program on sub-scales of stress, anxiety, and depression in the two groups. Note. Compared with the control group, * denotes *p* < 0.05, ** denotes *p* < 0.01. Compared with pre-test, # denotes *p* < 0.05, ## denotes *p* < 0.01.

As shown in Table 5, the results revealed that significant differences were observed in both the control group (*p* < 0.05) and the experimental group (*p* < 0.01). Figure 2a–c illustrated the trend of reduction in stress, anxiety, and depression for the two groups, respectively. The experimental group had a more noticeable reduction of stress, anxiety, and depression compared to the control group, which indicated that the program had a positive influence on the mental health of parents of children with ASD.

#### 3.1.2. Effects of Online Parent–Child Physical Activity Program on PSI-4-SF

The Parenting Stress Index-Fourth Edition-Short Form (PSI-4-SF) was also conducted to assess whether there were possible changes in mental health of the participants. As shown in Table 6, there were significant differences in terms of time: F = 137.995b, *p* < 0.01, and time × group interaction: F = 57.741b, *p* < 0.01. The results of the multivariate tests showed that the time factor had significant effects (*p* < 0.01) on PSI-4-SF over time, and the interaction between testing intervals (pre-test and post-test) and groups (experimental and control groups) also showed a statistically significant difference (*p* < 0.01)

As reported in Table 7, there were no significant differences in the overall PSI-4-SF score between the two groups in the pre-test (*p* < 0.05), while there were significant differences between the two groups in overall PSI-4-SF scores (*p* < 0.05) after the 10-week parent–child physical activity program, and even more significant changes were found in the experimental group, compared with the control group.

As reported in Table 8, the results revealed that there were significant differences from pre- to post-test in the experimental group (*p* < 0.01), but no significant differences were observed across the two measures (before/after) in the control group. Therefore, the results indicated that the web-based physical activity program had a positive effect on relieving parenting stress of parents of children with ASD, and it reduced the overall scores in PSI-4-SF.

**Figure 3:**
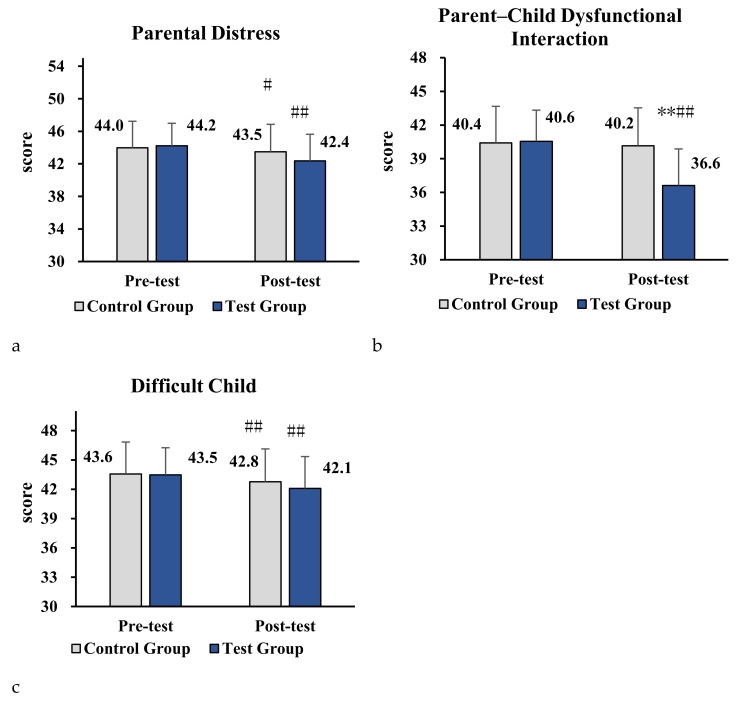
(**a–c**) Impact of parent–child physical activity program on sub-scales of parental distress, parent–child dysfunctional interaction, and child difficulty in the two groups. Note. Compared with the control group, * denotes *p* < 0.05, ** denotes *p* < 0.01. Compared with pre-test, # denotes *p* < 0.05, ## denotes *p* < 0.01.

As shown in Figure 3a–c, a significant reduction was found in parent–child dysfunctional interaction for the experimental group compared with the control group, which had a statistically significant effect (*p* < 0.01). These results also showed that there were significant differences in parental distress, parent–child dysfunctional interaction, and child difficulty for the experimental group across time (i.e., from pre- to post-test).

### 3.2. Effects of Parent–Child Physical Activity Intervention Program on Quality of Life

To determine the effects of intervention on quality of life on parents of children with ASD, the WHO Quality of Life Assessment-26 (QOL-26) was employed to examine the comparison outcomes. There were significant differences in terms of time: F = 48.608b, *p* < 0.01, and time × group interaction: F = 0.063b, *p* > 0.01, as reported in Table 9. The results of the multivariate tests showed that the time factor had a significant effect (*p* < 0.01) on QOL-26 over time, and the interaction between testing intervals (pre-test and post-test) and groups (experimental and control groups) showed no statistically significant difference (*p* > 0.01)

As reported in Table 10, before the intervention, there were no significant differences between the two groups (*p* > 0.05); furthermore, after the 10-week intervention, no significant differences were observed in overall QOL-26 between the experimental and control groups (*p* > 0.05).

As shown in Table 11, a paired samples *t*-test was conducted to determine whether a significant difference existed at two time points for the within-group comparison. The results revealed that there were significant differences from pre- to post-test in the experimental and control groups (*p* < 0.01).

**Figure 4:**
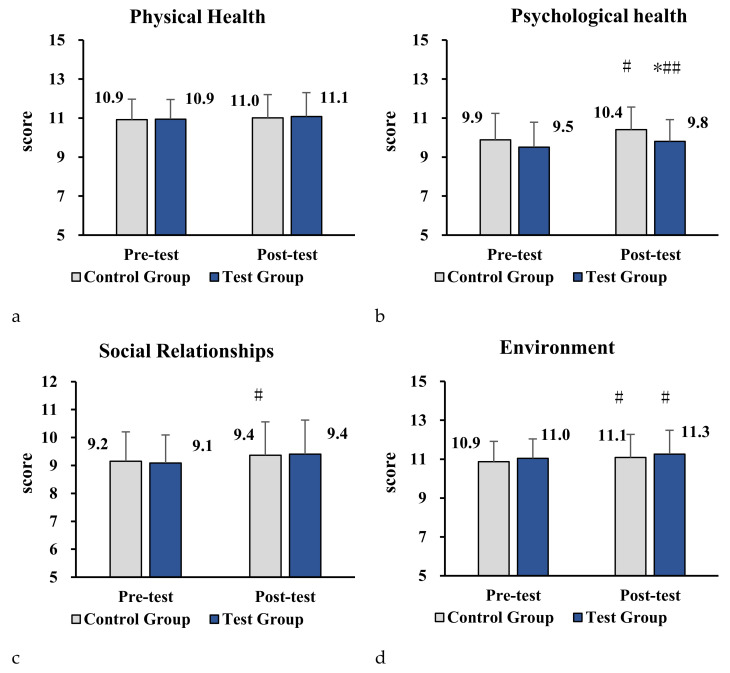
(**a–d**) Impact of parent–child physical activity program on sub-scales of physical health, psychological health, social relationships, and environment in the two groups. Note. Compared with the control group, * denotes *p* < 0.05, ** denotes *p* < 0.01. Compared with pre-test, # denotes *p* < 0.05, ## denotes *p* < 0.01.

We also evaluated whether the program demonstrated influences in the four sub-areas of the WHOQOL-26 between the experimental and control groups across pre/post testing intervals. Figure 4a–d display the trends in the sub-scales of WHOQOL-26 for the experimental group and control group. As shown in Figure 4a–d, a greater reduction was observed in the item of psychological health for the experimental group over time, compared with control group (*p* < 0.05). These findings demonstrated that the parent–child physical activity program significantly improved the psychological health sub-scale of WHOQOL-26 after the 10-week intervention.

## 4. Discussion

We investigated the effects of a 10-week web-based parent–child physical activity program on the mental health of parents of children with ASD. To our knowledge, this study may be the first randomized controlled trial involving a parent–child dyadic physical activity program intervention specifically designed to address the mental health of parents of children with ASD in a web-based context. The results of the present study provide evidence that web-based parent–child physical activity is effective in enhancing the mental health of parents of children with ASD, and also confirmed the potential effects of web-based parent–child physical activity as a functional and effective approach for parents of children with ASD.

Empirical evidence has shown that raising a child with ASD can be a stressful experience for parents, and parents raising children with ASD tend to experience even higher levels of stress and mental health difficulties than parents of children without ASD [5,9,47]. The mental health of parents includes their thoughts, emotional regulation, and behaviors, which reflect the psychological or biological processes underpinning their mental functioning. Based on previous studies, parental involvement during interventions in children with ASD is critical and essential for children’s development and improvement. From the existing literature in the field, however, most of the studies have focused on children as the main subjects, and the research evidence on involving parents of children in the rehabilitation process has been limited, whereas parents and their child with ASD, as a dyadic group participating in activities together during the intervention, requires further investigation. Therefore, the present study addresses the limitations of previous studies, in terms of the lack of focus on improving the mental health of parents of children with ASD.

The study examined the changes in mental health of parents of children with ASD through the DASS-21, PSI-4-SF, and WHOQOL-26 instruments after a 10-week online parent–child physical activity intervention. Our findings confirmed the significant differences in DASS-21 and PSI-4-SF for the experimental group, when compared with the control group across time, and the significant reduction in overall scores in DASS-21 and PSI-4-SF after the intervention. There was no significant difference in overall WHOQOL-26 scores between the two groups, which may be because the QOL reflects multi-dimensional aspects of life, and the intervention program promotes only several dimensions with a 10-week duration. We speculate that a longer intervention may be helpful to improve other aspects of QOL and achieve significant changes in overall QOL.

Among the three sub-scales of DASS-21 and the PSI-4-SF, they all had statistically significant differences in terms of the stress, anxiety, and depression after the intervention, indicating the positive influence on relieving the stress of parents of children with ASD through participating in the parent–child physical activity program. One possible explanation for the effectiveness of the intervention is the nature of this web-based family-centered program to provide great opportunities for the parent and child to interact with each other, and the step-by-step structured approach easily engages parents and their children in the physical activity sessions together. The evidence obtained clearly indicated that using a family-centered parent–child program targets the core symptoms of children with ASD and has a dramatic impact on children’s weakness to interact and communicate with others, including their parents, which might be major reasons resulting in the psychological anxiety and depression of the parents.

The results of this study confirmed that the parent–child physical activity program can be viewed as a natural environment for building relationships between parents and their children with ASD. The constructed activity program involves co-participation physical activities that naturally engage the parent–child pair, which is crucial for the family to increase parent–child communication, time spent together, and enjoyment of physical activity. During each session, the parents have great opportunities to experience interactions with their children through different kinds of activities that were not impossible before. Such enjoyable experiences greatly increase dyadic shared pleasure, as well as parental self-efficacy and motivation when interacting with their children, while reducing parenting stress and enhancing mental health for both parents and their children.

It has been shown that the mental health of a family unit and family members has a significant positive correlation with their communication and interaction [48], and it may explain the effectiveness of the current intervention in improving the mental health of parents of children with ASD through increased interaction and communication during participation in the parent–child physical activity program. Specifically, the program provides a family-based activity environment with warm, interactive, and cohesive styles that promote the shared participation of parents and children with ASD. Parents can influence their children’s physical activity participation through role-modeling (being active themselves), material support (financial, logistic, co-participation), and encouragement [49]. Parental role-modeling and support for physical activity benefit children with ASD and can encourage children to be more active in their interactions with others [50]. After constant repetition and practice, it may create a constructive interaction environment, which leads to a virtuous cycle. When the parents see their children’s progress, it inevitably leads to reduced psychological pressure. Therefore, we believe that structured parent–child dyadic physical activity can lead to a natural environment that promotes the relationship between parents and their children within a parent–child family context.

Another possibility for the improvement of mental health through the intervention was the essential characteristics of web-based technology supporting the physical activity program. During the COVID-19 lockdown, web-based programs have become popular and necessary, providing an engaging way to easily involve children with ASD and parents in physical activity programs. The web-based parent–child physical activity program provided more opportunities to cope with stress through the use of different kinds of modern scientific and technological innovations, such as video games, an online gym, and a live course, which all provide a rich communicative platform including a chatroom, instant messenger, bulletin boards, art activities, contests, and more, in order to support the interaction and socialization of the participants. With this advanced technology, the significant results of the present study might serve as good practical experience for those special schools and rehabilitation centers where most of the physical activity programs are conducted in the institution, which typically target children with ASD only, without involving their family members. In particular, the web-based parent–child program can assist those parents who are struggling between work and family, and who face greater difficulty in attending school-based parent–child exercise programs. Hence, there is a need to develop a set of web-based parent–child exercise programs for parents and children, allowing them to get active in a convenient setting, as a home-based environment (as a treatment setting) is more comfortable for parents and their children. Therefore, it could be implemented with more joy for the family members.

Many previous studies have indicated the importance of peer support and group practice [51,52]; as such, we scheduled two sessions of group exercises and live courses, conducted online through Zoom and Tencent Live. The participants also had opportunities to watch other children and families practicing and performing. We believe that this setting may be beneficial to the parents of children with ASD, as Zoom provides them with opportunities to communicate with other parents who are going through similar experiences and difficulties. Through interaction and communication with other parents, these parents could release their stress and improve their mental health. Our findings confirm that family-centered, collaborative, and web-based parent–child physical activity approaches can establish better parent–child relationships for communication, reduce parental stress and improve mental health, and eventually improve the quality of life of families that have children with ASD.

There are some limitations and future directions that we need to consider in future studies. First, the follow-up period was relatively short, and the changes in mental health may be unstable. It is necessary to observe the effects of the parent–child physical activity program on the mental health of parents of children with ASD over a longer period of intervention. Second, although we demonstrated, in the current study, that the parent–child physical activity program had a positive influence on the mental health of parents of children with ASD, it may be worthwhile to further explore whether there is a relationship between the effectiveness of the program and other family members, such as their siblings and other people who live with them. The potential contributions of the parent–child physical activity program toward parental mental health, health-related fitness, and family well-being will be another important direction for future research.

## 5. Conclusions

The findings of the present study were in line with the results of previous studies, and the results of the present study demonstrated that the web-based structured parent–child physical activity program can significantly improve the mental health of the parents of children with ASD. The program strongly demonstrated a positive influence on the overall DASS-21 and PSF-4-SF scores, and significantly enhanced the sub-scales of three instruments in the areas of depression, anxiety, stress, parental distress, parent–child dysfunctional interaction, child difficulty, and psychological health. The findings of the present study also indicated that addressing the mental health of parents with children with ASD is necessary. We believe that the described web-based family program can be adopted by schools and rehabilitation centers as an alternative approach to enhance parent–child relationships, as well as to improve the mental health of parents of children with ASD.

## Figures and Tables

**Table 1:** Demographic data of the participants.

Variables	Experimental Group	Control Group	Total	F/t/${𝝌}^{2}$	*p*-Value
Participant—children					
Age, y, mean (SD)	6.0 (1.7)	5.8 (1.5)	5.9 (1.6)	0.357	0.722
Gender, n, M/F					
Participant—parents					
Age, y, mean (SD)	33.8 (3.9)	33.4 (4.2)	33.6 (4.0)	0.417	0.678
Gender, n, M/F	11/26	11/27	22/53	0.006	0.941
Number of children in the family				−0.129	0.898
One child	22	24	46		
Two children	16	13	29		
Parent education level				0.457	0.796
Secondary education	7	9	16		
University education	27	25	52		
Post-graduate and above	3	4	7		
Parent employment				0.829	0.661
Employed	12	13	25		
Housewives/househusbands	25	25	50		

**Table 2:** Parent–child physical activity intervention program.

Parts	Time (min)	Content	Way
Warm-up activities	10	Parent–child warm-up exercises (make up their own moves to music); Walking Lunges; Arm Circles or Swings.	Teaching video
Parent–child exercises	20	Jumping jack; Side Hops; Parent–child yoga; Play ball exercises and games with their teachers.	Live course and teaching video
Play-based exercises	20	Interactive exercise through video games (Run-ning; Tennis; Fruit Slice).	Motion sensing game
Cool-down and reward activities	10	Stretching and relaxation exercises; High five and clap; Sing goodbye songs together.	Live course and teaching video

**Table 3:** Multivariate Tests.

Index	Effect	Pillai’s Trace	F	Hypothesis df	Error df	Sig.	Partial η^2^	Sample Power
Overall DASS-21	Time	0.670	147.937	1.000	73.000	0.000	0.670	1.000
	Time X group	0.592	106.032	1.000	73.000	0.000	0.592	1.000
Stress	Time	0.576	99.023	1.000	73.000	0.000	0.576	1.000
	Time X group	0.482	67.826	1.000	73.000	0.000	0.482	1.000
Anxiety	Time	0.475	66.023	1.000	73.000	0.000	0.475	1.000
	Time X group	0.419	52.538	1.000	73.000	0.000	0.419	1.000
Depression	Time	0.533	83.165	1.000	73.000	0.000	0.533	1.000
	Time X group	0.442	57.821	1.000	73.000	0.000	0.442	1.000

**Table 4:** Comparison of DASS-21 across two intervals between two groups.

Variables	Control Group	Experimental Group	t	df	*p*-Value	d	Sample Power
Pre-Test							
Overall DASS-21	42.11 ± 9.96	42.55 ± 10.92	−0.184	73	0.854	0.043	0.054
DASS-21 depression	14.38 ± 3.49	14.66 ± 3.74	−0.334	73	0.739	0.077	0.063
DASS-21 anxiety	13.11 ± 3.65	13.13 ± 3.92	−0.027	73	0.979	0.005	0.050
DASS-21 stress	14.62 ± 3.59	14.76 ± 3.92	−0.163	73	0.871	0.037	0.053
Post-Test							
Overall DASS-21	41.51 ± 9.23 ^#^	35.39 ± 9.11 ** ^##^	2.889	73	0.005	0.667	0.814
DASS-21 depression	14.14 ± 3.27	12.08 ± 3.21 ** ^##^	2.750	73	0.008	0.636	0.775
DASS-21 anxiety	13 ± 3.32	11.24 ± 3.27 * ^##^	2.319	73	0.023	0.534	0.626
DASS-21 stress	14.38 ± 3.34 ^#^	12.08 ± 3.49 ** ^##^	2.912	73	0.005	0.673	0.821

Note. Compared with the control group, * denotes *p* < 0.05, ** denotes *p* < 0.01. Compared with pre-test, ^#^ denotes *p* < 0.05, ^##^ denotes *p* < 0.01.

**Table 5:** Pre-/post-comparison of DASS-21 for two groups.

	Pre-Test	Post-Test	Difference	t	df	*p*-Value	d	Sample Power
Control Group	42.11 ± 9.96	41.51 ± 9.23 ^#^	0.595	2.507	36	0.017	0.412	0.615
Experimental Group	42.55 ± 10.92	35.39 ± 9.11 ** ^##^	7.158	12.237	37	0.000	1.985	1.000

Note. Compared with the control group, * denotes *p* < 0.05, ** denotes *p* < 0.01. Compared with pre-test, ^#^. denotes *p* < 0.05, ^##^ denotes *p* < 0.01.

**Table 6:** Multivariate Test.

Index	Effect	Pillai’s Trace	F	Hypothesis df	Error df	Sig.	Partial η^2^	Sample Power
Overall PSI-4-SF	Time	0.654	137.995	1.000	73.000	0.000	0.654	1.000
	Time X group	0.442	57.741	1.000	73.000	0.000	0.442	1.000
Parental Distress	Time	0.427	54.444	1.000	73.000	0.000	0.427	1.000
	Time X group	0.202	18.452	1.000	73.000	0.000	0.202	0.989
Dysfunctional Interaction	Time	0.507	74.937	1.000	73.000	0.000	0.507	1.000
	Time X group	0.445	58.548	1.000	73.000	0.000	0.419	1.000
Difficult Child	Time	0.422	53.361	1.000	73.000	0.000	0.422	1.000
	Time X group	0.049	3.740	1.000	73.000	0.057	0.049	0.48

**Table 7:** Comparison of PSI-4-SF across two intervals between two groups.

Variables	Control Group	Experimental Group	t	df	*p*-Value	Effect Size	Sample Power
Pre-Test							
Overall PSI-4-SF	127.95 ± 6.61	128.24 ± 6.6	−0.191	73	0.849	0.044	0.054
Parental Distress	43.97 ± 3.26	44.21 ± 3.37	−0.310	73	0.757	0.072	0.061
Parent–Child Dysfunctional Interaction	40.41 ± 3.19	40.55 ± 3.28	−0.197	73	0.844	0.043	0.054
Difficult Child	43.57 ± 3.24	43.47 ± 3.24	0.125	73	0.901	0.031	0.052
Post-Test							
Overall PSI-4-SF	126.41 ± 5.62	121.05 ± 5.69 ** ^##^	4.095	73	0.000	0.946	0.982
Parental Distress	43.49 ± 2.78	42.37 ± 3.27 ^##^	1.595	73	0.115	0.369	0.352
Parent–Child Dysfunctional Interaction	40.16 ± 3.01	36.61 ± 2.93 ** ^##^	5.185	73	0.000	1.195	0.999
Difficult Child	42.76 ± 3.2	42.08 ± 3.13 ^##^	0.927	73	0.357	0.215	0.151

Note. Compared with the control group, * denotes *p* < 0.05, ** denotes *p* < 0.01. Compared with pre-test, ^#^ denotes *p* < 0.05, ^##^ denotes *p* < 0.01.

**Table 8:** Pre/post comparison of PSI-4-SF for two groups.

	Pre-Test	Post-Test	Difference	t	df	*p*-Value	d	Sample Power
Control Group	127.95 ± 6.61	126.41 ± 5.62	1.541	4.800	36	0.100	0.789	0.996
Experimental Group	128.24 ± 6.6	121.05 ± 5.69 ** ^##^	7.184	10.839	37	0.000	1.758	1.000

Note. Compared with the control group, * denotes *p* < 0.05, ** denotes *p* < 0.01. Compared with pre-test, ^#^ denotes *p* < 0.05, ^##^ denotes *p* < 0.01.

**Table 9:** Multivariate Tests.

Index	Effect	Pillai’s Trace	F	Hypothesis df	Error df	Sig.	Partial η^2^	Sample Power
Overall QOL-26	Time	0.400	48.608	1.000	73.000	0.000	0.400	1.000
	Time X group	0.001	0.063	1.000	73.000	0.802	0.001	0.057
Physical Health	Time	0.052	4.042	1.000	73.000	0.048	0.052	0.510
	Time X group	0.002	0.142	1.000	73.000	0.708	0.002	0.066
Psychological Health	Time	0.128	10.727	1.000	73.000	0.002	0.128	0.898
	Time X group	0.000	0.008	1.000	73.000	0.931	0.000	0.051
Social Relationships	Time	0.097	7.810	1.000	73.000	0.007	0.097	0.788
	Time X group	0.004	0.274	1.000	73.000	0.603	0.004	0.081
Environment	Time	0.455	60.904	1.000	73.000	0.000	0.455	1.000
	Time X group	0.062	4.824	1.000	73.000	0.031	0.062	0.582

**Table 10:** Between-group comparison for WHOQOL-26 across two intervals.

Variables	Control Group	Experimental Group	t	df	*p*-Value	Effect Size	Sample Power
Pre-test							
Overall QOL-26	40.83 ± 2.86	40.58 ± 2.74	0.376	73	0.708	0.087	0.067
Physical Health	10.92 ± 1.05	10.95 ± 1.19	−0.110	73	0.913	0.027	0.051
Psychological Health	10.86 ± 1.49	11.04 ± 1.45	−0.501	73	0.618	0.122	0.082
Social Relationships	9.15 ± 1.94	9.09 ± 2.01	0.144	73	0.886	0.030	0.052
Environment	9.89 ± 1.35	9.51 ± 1.16	1.295	73	0.199	0.302	0.252
Post-test							
Overall QOL-26	41.87 ± 2.84 ^##^	41.55 ± 2.93 ^##^	0.473	73	0.637	0.109	0.076
Physical Health	11.01 ± 1	11.08 ± 1.22	−0.275	73	0.784	0.063	0.058
Psychological Health	10.41 ± 1.27 ^#^	9.8 ± 1.12 ^##^ *	2.179	73	0.033	0.510	0.585
Social Relationships	9.37 ± 1.87 ^#^	9.4 ± 1.81	−0.080	73	0.936	0.016	0.051
Environment	11.08 ± 1.33 ^#^	11.26 ± 1.6 ^#^	−0.535	73	0.594	0.122	0.082

Note. Compared with the control group, * denotes *p* < 0.05, ** denotes *p* < 0.01. Compared with pre-test, ^#^ denotes *p* < 0.05, ^##^ denotes *p* < 0.01.

**Table 11:** Pre/post comparison of WHOQOL-26 for two groups.

	Pre-Test	Post-Test	Difference	t	df	*p*-Value	d	Sample Power
Control Group	40.83 ± 2.86	41.87 ± 2.84 ^##^	−1.041	−8.310	36	0.000	1.366	1.000
Experimental Group	40.58 ± 2.74	41.55 ± 2.93 ^##^	−0.969	−3.770	37	0.001	0.612	0.961

Note. Compared with the control group, * denotes *p* < 0.05, ** denotes *p* < 0.01. Compared with pre-test, ^#^ denotes *p* < 0.05, ^##^ denotes *p* < 0.01.

## Data Availability

The data presented in this study are available on request from the corresponding or first author.
